# Spanish and Swedish eldercare managers’ influence on employees

**DOI:** 10.1108/IJWHM-02-2018-0014

**Published:** 2018-10-01

**Authors:** Maria Nordin, Marina Romeo, Montserrat Yepes-Baldó, Kristina Westerberg

**Affiliations:** 1 Department of Psychology, Umeå University, Umeå, Sweden; 2 University of Barcelona, Barcelona, Spain

**Keywords:** Managerial commitment, Employee mental well-being, Employee perception of quality of care, Employee turnover intent, Managerial overcommitment, Type of organization

## Abstract

**Purpose:**

Hierarchical and flat organizational types are predominant in Spain and Sweden, respectively. To study how managers’ commitment and work overcommitment (WOC) affect employee well-being, and job perception in these different countries can shed insight on how to improve eldercare organization. The purpose of this paper was to study the association between eldercare employee exposure to managers’ commitment and WOC, and employee mental well-being and job perception and how these associations differed between Spain and Sweden.

**Design/methodology/approach:**

A questionnaire with validated questions on commitment, WOC, mental well-being and job perception, operationalized as the perception of quality of care and turnover intent, was sent out to eldercare managers and employees in Spain and Sweden. *t*-Tests, *χ*^2^ and linear regression were applied to study the associations and differences between the countries.

**Findings:**

Interaction analyses revealed that Spanish employees’ mental well-being and job perception were influenced by their managers’ commitment and WOC in that manager commitment improved and WOC impaired well-being and job perception. However, the Swedish eldercare employees were not influenced by their managers on these parameters.

**Practical implications:**

The impact of managerial commitment and WOC differed between employees in Spain and Sweden, possibly because the preconditions for leadership varied due to differences in organizational type.

**Originality/value:**

This study compares the managers’ impact on employee health and job perception in two countries with different organizational prerequisites. Moreover, managers’ commitment and WOC were estimated by the managers themselves and did not rely on the employees’ perception, which improved ecological validity.

One of the greatest social and economic challenges for Europe is the ageing population. A growing number of persons will be in need of pensions, care and services in a near future (European Commission, 2015). Moreover, many European countries report difficulties in both retaining and recruiting health care staff. Being part of Europe, both Spain and Sweden are expected to meet these increasing demands in eldercare. The countries represent the north and south of Europe with its similarities and differences. At the time of the start of the present study (2013), the aftermath of the 2008 crisis was still evident, foremost in Spain that was hit harder by the economic decline than Sweden. According to Eurostat, the rates of unemployment were 25 percent for the Spanish population and 8 percent for the Swedish and the countries at the time battled different problems. In Spain, high unemployment rates and few alternatives on the labor market led to feelings of being forced to stay in an organization, whereas in Sweden, there were, and still is, significant problems with recruitment and turnover rates in eldercare. Thus, the preconditions for conducting eldercare were different in the two countries, even if the demand on expanding was the same.

In order to meet these challenges, commitment of employees is crucial. Meyer and Herscovitch (2001, p. 301) define commitment as “a force (mind-set) that binds an individual to a course of action of relevance to one or more targets.” Commitment has been not only negatively associated with turnover intentions (Wong and Spence Laschinger, 2015) and stress (Meyer *et al.*, 2002; Glazer and Kruse, 2008) but also positively associated with intention to stay (Brown *et al.*, 2013), employee performance and organizational citizenship behaviors (Meyer *et al.*, 2002) as well as well-being (Meyer and Maltin, 2010; Clausen *et al.*, 2015). Many studies address employee commitment. However, leader commitment has only recently been shown to be a key to both positive outcomes such as promoting employee engagement and performance (Gutermann *et al.*, 2017) and to more negative ones, such as burnout (Huang *et al.*, 2016). In general, leadership behavior is associated with employee satisfaction and affective health (Skakon *et al.*, 2010). A stressed out leader may display behaviors that can contribute to stress among the employees (Huang *et al.*, 2016), whereas a committed leader’s behavior can lead to employee engagement.

Another concept seemingly related to commitment is work overcommitment (WOC). However, stemming from a different theoretical background, WOC refers to a set of attitudes, behaviors and emotions that reflect excessive striving (Siegrist, 2017) and being so committed to work that it is hard to let it go when it is time to recover. WOC is a central concept in the Effort-Reward Imbalance model as it represents a personal pattern of coping which is considered to be vulnerable to stress (Siegrist *et al.*, 2004). Although this part of the theory has not been completely confirmed, WOC as such has been shown to be a risk factor of health complaints, such as sleep disturbances (Åkerstedt *et al.*, 2012). Managers have reported higher levels of WOC compared to other employees (Rystedt *et al.*, 2007). However, Feldt *et al.* (2013) argue that WOC is not solely a negative factor for managers, since WOC means high work engagement which may benefit the employees.

Managers play an important part in creating the work environment and in making it sustainable and attractive to the employees. Leaders are mediators of organizational support, which, in turn, is important for employee commitment (Guest *et al.*, 2010). Support from the manager is a potent buffer against stress among employees (Skakon *et al.*, 2010), whereas an invisible leadership, such as laissez-faire, is associated with mental distress and poor well-being (Skogstad *et al.*, 2007). If the manager is committed to work and have the organizational preconditions (such as spatial proximity to the employees, a small or large enough group (control span) to lead, etc.) to show this commitment, it will likely reflect positively on both the work environment and employee health. However, if commitment turns into an unhealthy WOC that causes stress for the manager, the relationship may become adverse.

Spain and Sweden differ not only in societal aspects but also in ideas on how to organize. In comparison to European managers on average, Spanish managers promote less influence by employee representatives and show a higher need for control (Munduate *et al.*, 2015). In contrast, managerial ideas of New Public Management have led to wider spans of control in Sweden (Björk *et al.*, 2013). In hospital care, nurses report larger dissatisfaction with management in Sweden, but higher equality in relation to other top-level executives compared to Spain (Aiken *et al.*, 2013). In short, Spanish organizations are hierarchically characterized whereas the characteristics of the Swedish ones are shared responsibilities and thus a flatter organization type. In other words, power distance (i.e. the degree people who are not in power accept that power is spread unequally; Hofstede, 1980, 2001) is high in Spain but low in Sweden.

Given the different preconditions of the two countries, along with the above discussion on commitment and WOC, the aim of this study was to investigate differences between Spain and Sweden regarding managers’ commitment and WOC and its association to employee mental well-being, perception of performance in the form of quality of care (QoC), and turnover intent. We hypothesize that:

H1. There is a difference between Spanish and Swedish employees’ exposure to managers’ commitment and WOC, as well as their mental well-being, perception of QoC and turnover intent.

H2. There are differences between Spain and Sweden regarding how employees’ exposure to managers’ commitment is associated with employees’ mental well-being, perception of QoC and turnover intent.

H3. There are differences between the countries in how the employees’ exposure to managers’ WOC is associated with employees’ mental well-being, perception of QoC and turnover intent.

## Method

### Procedure and sample

A cross-sectional study in eldercare organizations in Spain and Sweden is used for the purposes of this study. Data were collected by inviting managers and employees in eldercare organizations in the two countries to fill out a questionnaire on, among other themes, commitment, WOC, mental well-being, perception of QoC and turnover intent.

In Spain, four residential homes in four municipalities in Catalonia were selected by convenience and offered to participate. All organizations accepted. The organizations are characterized by hierarchical structures with top-, middle- and first-line managers. The questionnaires were distributed by the researchers during spring 2015 and the response rate was 88 percent (353/400; 46 managers and 307 employees).

The participating organizations in Sweden were recruited from a manager training program. The project was presented to 20 managers who got the opportunity to sign up if they were interested to participate together with their staff. This convenience sample includes nine workplaces from eight municipalities in the mid-north of Sweden. They represent both residential care (82.7 percent) and home help services (17.3 percent). The Swedish organizations are characterized by being flat and represented by first-line managers with responsibility for budget and staff at their unit. The questionnaires were distributed by the managers during spring of 2015 and 2016. Each participant got a prepaid envelope in which they could send their answers to the researchers directly. The response rate was 54 percent (299/558; 13 managers and 286 employees).

The samples are similar in aspects such as age and gender distribution (see Table I). However, the Spanish sample constitutes of proportionally more managers than the Swedish (13 vs 4.4 percent). Moreover, in the Spanish sample eight (17 percent) of the managers are men and in the Swedish sample one person (7.7 percent) is a male manager. Of the employees, 29 (11 percent) are men in the Spanish sample. In the Swedish sample, the corresponding figure is 11 (6.7 percent). Thus, the proportion of men is larger in Spain, both as managers and as employees.

### Questionnaires

#### Background variables

Sociodemographic data in the form of country (Spain or Sweden), age and sex were collected as was information on having a managerial position or not.

#### Independent variables

Data from managers’ reply on the commitment and WOC scales were used as independent variables. Commitment was assessed by the commitment questions in the identification-commitment inventory (ICI) (Romeo *et al.*, 2011), which includes 12 items with a five-point response scale ranging from “hardly ever” to “very often.” The ICI includes four dimensions: need commitment (e.g. “I would not recommend any family member or friend that they should work in this organization”); exchange commitment (e.g. “An important reason why I continue working in this organization is that I don’t feel that other organizations can offer me better compensation”); value commitment (e.g. “I feel that there is a big similarity between my personal values and those of this organization”); and affective (e.g. “The success of my organization is my success”). Cronbach’s *α*s were 0.69 for the need scale, 0.69 for the exchange scale, 0.70 for the affective scale and 0.78 for the value scale. To obtain a general commitment score a mean was calculated including all items, after reversing the need commitment items (Cronbach’s *α* 0.93). The WOC scale (Siegrist *et al.*, 2004) was used to measure overcommitment at work. The scale contains six items with a five-point response scale ranging from “very seldom or never” to “very often.” “People close to me say I sacrifice too much for my job” and “As soon as I wake up in the morning, I think about work” are examples of items included in the scale. The mean of the six items was used for the purposes of this study. Cronbach’s *α* was 0.83.

#### Dependent variables

Data from employees’ replies on mental well-being and job perception, operationalized as QoC and turnover intent, were used as dependent variables. To measure mental well-being the General Health Questionnaire-12 items was used. This is a one-dimensional instrument developed originally by Goldberg (1972). The participants are asked to indicate how often they have experienced symptoms that reflect their mental well-being in the last four weeks. An example of item is “How often have you felt constantly under stress?” Replies are given on a four-point scale ranging from “much less than usual” to “more than usual.” Thus, a higher score indicates a poorer mental health. Mean scores were calculated and Cronbach’s *α* was 0.86.

QoC (Westerberg and Tafvelin, 2014) is a five-item instrument that forms a measure of QoC as assessed by the staff. The five response alternatives range from “very seldom or never” to “very often or always.” Examples of items are “At my workplace I experience that enough consideration is taken to the users’ opinions and wishes” and “In general, I am satisfied with the quality of care provided at this workplace.” Mean scores across the five items were calculated and Cronbach’s *α* was 0.82.

Turnover intent was measured by a single item “I am going to look for another job in the coming year” with the response scale 1–5, ranging from “strongly disagree” to “strongly agree” (Hom *et al.*, 1984).

### Statistical analyses

To calculate the differences between Spain and Sweden in commitment, WOC, employees’ mental well-being, perception of QoC and turnover intent, independent sample *t*-tests were performed. To calculate the differences between the two countries’ proportion of men, women and managers, *χ*^2^ analyses were used.

To study the employees’ exposure to managers’ commitment and WOC, a variable was created where each employee was assigned with their respective manager’s commitment scores and WOC scores. For those who had several managers (only relevant to the Spanish sample), an average across the managers was calculated. Thereafter, the variables were *z*-transformed and interaction terms were created by multiplying them with the variable for country, coded 1 for Spain and 2 for Sweden. Subsequently, linear regression analyses were performed by including the interaction terms along with their respective main effect variables and the dependent variables mental well-being, QoC and turnover intent. Significant interaction effects prompted *post hoc* linear regression analyses for Spain and Sweden, respectively, by using the commitment and WOC variables as independent variables and mental well-being, QoC and turnover intent as the dependent variables. In a second step, sex and age were used to check for confounding. *α* was set to 0.05.

## Results

The results show that there were significant differences between Spain and Sweden in all studied variables (see Table II). Spanish employees were exposed to a significantly higher level of commitment but a significantly lower level of WOC from their managers compared to the Swedish. Also, the Spanish employees reported better mental well-being, they perceive to give a higher QoC and they reported a lower turnover intent than the Swedes.

Analyzing the separate dimensions included in the commitment scale, i.e., need, exchange, affective and value, showed the same pattern as did the global scale. Therefore, only the global scale was used in further analyses.

### Commitment

A significant interaction effect was found between managers’ commitment and country in the association with mental well-being, QoC and turnover intent, indicating that the Spanish and Swedish employees were affected differently by their managers’ commitment (see Table III, upper part). A *post hoc* analysis revealed that managers’ commitment increased employee perception of QoC and decreased mental well-being and turnover intent significantly in their employees in Spain. The *β* values were only marginally changed when adjusting for sex and age. Moreover, the explained variance for the original models was low (4–6 percent). However, for turnover intent, it increased when adjusting for gender and age. A closer investigation showed age to be the influencing factor (see Table IV, upper part).

*Post hoc* results for the Swedish sample (see Table IV, upper part) not only showed non-significant results, the *β* values were also very close to 0, indicating that managers’ commitment had barely any effect on either mental well-being, perception of QoC or turnover intent. The explained variance was close to 0 in all the original models. However, sex and age increased it also here and again age was the explaining factor. Age was negatively associated with turnover intent in all the models that included commitment and turnover intent.

### Work overcommitment

Regarding WOC, there was a significant interaction effect between managers’ WOC and country on QoC and turnover intent (see Table III, lower part). *Post hoc* analyses showed that managers’ WOC decreased the perception of QoC in both the Spanish and Swedish samples, but to a larger extent in the Spanish sample. It also revealed WOC to be associated with an increase in turnover intent in the Spanish sample. In the Swedish sample, there was no relationship between managers’ WOC and turnover intent (see Table IV, lower part). Again, the explained variances were very low for the original models but increased when sex and age were added. Age was the factor that increased the explained variance. Moreover, age was negatively associated with turnover intent.

## Discussion

The aim of this study was to investigate if managers’ commitment and WOC were differently associated with employees’ mental well-being, perception of QoC and turnover intent in Spain and Sweden. The first hypothesis, that there is a difference between Spain and Sweden in employees’ exposure to managers’ commitment and WOC, was fulfilled since Spanish employees reported to have managers who report more organizational commitment and less WOC than the Swedish employees did. At the same time, Spanish employees reported better mental well-being, that they provided a higher QoC, and had less intentions to leave their organization than the Swedes. The second and third hypotheses were also fulfilled since managers’ commitment differed in its association with employee mental well-being, perception of QoC and turnover intent between Spain and Sweden. The same pattern was found for managers’ WOC with the exception that there was no difference between the countries in the association with mental well-being.

### Commitment

The Spanish sample in this study was characterized by a larger proportion of managers than the Swedish one, which implies that the Spanish managers have a smaller control span. Also, in the Spanish sample, exposure to managers’ commitment was associated with more mental well-being and quality of care along with less turnover intent. Given the positive associations between managers’ behavior and employee health confirmed by many studies (Skakon *et al.*, 2010), it is not unlikely that managers who commit to an organization display behaviors that inspire employees. This, in turn, may be reflected in the employees’ well-being, performance and wanting to stay. In fact, a recent study showed that leaders’ work engagement increased employees’ performances and decreases their turnover intent (Gutermann *et al.*, 2017), and the authors argued the exchange between leaders and followers to be the link. Thus, leaders’ can have a positive impact on their followers by showing organizational commitment. Interestingly, no such association was seen in the Swedish sample in the present study. First, the Swedes were exposed to a lower mean of managers’ commitment and second, there was hardly any association between the Swedish managers’ commitment and the employees’ mental well-being, perception of QoC or turnover intent since the *β* values were close to 0. In order for leaders and members to exchange experiences, values and commitment, there must be adequate prerequisites. It can be argued that the Swedish managers do not have that. In Sweden, last decades’ development of New Public Management has contributed to larger groups of employees in eldercare (up to 100 employees per manager; Astvik, 2003). Moreover, the employees are scattered in different eldercare settings, making it difficult for the managers and employees to meet on a daily basis to exchange valuable information. An inevitable consequence is that the managers’ availability and precondition to show commitment and to engage in the employees are limited (Astvik, 2003) and the leadership can in worst case, turn into a laissez-faire-like style. Also, more employees result in a larger work load and potentially less managerial control (Wallin *et al.*, 2014).

### Work overcommitment

Not only can managers’ commitment affect the employees’ positively. Huang *et al.* (2016) showed that burnout in managers lead to burnout in their followers too by depleting personal resources such as self-efficacy, self-esteem and optimism. Committed individuals may become overcommitted to work and experience role overload, i.e., having too many responsibilities and expected activities to perform in relation to their ability and time. In fact, Bolino and Turnley (2005) found that being highly committed to the organization was associated with both role overload and work-related stress. Flat organizations with their diffusion of responsibilities and large control spans may contribute to experiences of such overload. Thus, WOC may hypothetically increase the risk of role overload, especially in flat organizations.

It has been hypothesized that WOC is associated with stress, and indeed a meta-analysis by Eddy *et al.* (2018) confirmed its association with biomarkers such as the hypothalamic-pituitary-adrenal axis. Stress increases the risk of, for instance, irritation and poor judgment. Irritated managers who make bad decisions may very well affect the employees negatively and therefore the results of a decrease in Spanish employees’ perception of QoC and increase in their intention to leave due to managers’ WOC can be expected. This is also in line with much of previous results showing that managerial stress is related to employees’ affective health (Skakon *et al.*, 2010; Huang *et al.*, 2016). There was no country-specific difference in how managers’ WOC affected mental well-being in the present study, which may indicate a healthy worker effect in the Spanish sample. In the Swedish sample, managers’ WOC decreased the perception of QoC in their employees significantly, but to a very low degree (*β* =−0.08). Furthermore, there was no association between managers’ WOC and employee mental well-being or turnover intent in the Swedish sample. Again, this is an indication that the managers do not reach the employees. However, in this case it may be a good thing for the Swedes.

### Cultural differences

To understand differences between cultures, Hofstede’s multidimensional culture framework is often used (1980, 2001). An especially interesting dimension of the theory is power distance when it comes to management. Spain scores higher on power distance than Sweden in studies on general culture (e.g. Hofstede, 1980, 2001). Thus, Swedes are presumably less likely to adhere to more hierarchical organizational structures since that typically means more unequally spread power. Fisher (2014) shows that in cultures with low power distance, empowerment buffered against high workload and thus increased the possibilities for organizational commitment. Low power distance goes hand in hand with the idea of flat organizations and empowerment is one of the benefits of this type of organization. Taking this into consideration, implementation of more managers and more hierarchical structures in Swedish eldercare may meet resistance among employees. It is not unlikely that they would value the benefits and possibilities that come with flat organizations to a larger extent than the outcome variables investigated in this study.

### Implications for management

The present study shows that there are differences in how managers’ commitment and WOC affect employees in eldercare services in Spain and Sweden. Organizing in a way that makes sure that managers can provide their employees with a committed leadership will increase employees’ health and performance and decrease the want to leave the organization. The preconditions for the managers in the Spanish and the Swedish samples to perform their leadership differed. For instance, there were proportionally more managers in the Spanish sample, which is in line with the indications that Spanish organizations are more hierarchical than the Swedish ones. However, flat organizations, which are the predominant Swedish organization type, have often been promoted as they enhance empowerment and engagement among employees (Carney, 2004). Our study implies though that a flat organization type also can reduce the managers’ possibilities to affect employees by committing to work if, for instance, the span of control is too large. A flat organization type may therefore limit the managers’ prerequisites to lead, inspire and support the employees’ so that they in turn can engage and commit to their work, provide good care and create stability by remaining in the organization and stay healthy.

### Strengths and limitations

This study’s merits lie in the fact that responses from two different countries, which are dominated by two different organizational structures, were investigated with the same method and validated questionnaires with good internal reliabilities. Thus, they can be compared. A limitation is that the research is cross-sectional and therefore temporal sequence cannot be established. However, it seems less likely that employee mental well-being, perception of QoC and turnover intent precede managers’ commitment than the other way around, since the managers’ commitment was not a reflection of the employees’ perception but of the managers themselves in this study. Nevertheless, since the means of the managers’ responses are point estimates, the independent variables may still be inflated by reporting bias and mood. This is of course also true for the dependent variables.

Convenience sampling increases the risk of sampling error. Thus, the samples used in this study may not be representative. The use of several communities increased the possibilities for representativeness though. Also, the fact that the Swedish managers distributed the questionnaires when recruiting participants may have deterred some from participating. However, since the filled out questionnaires were to be sent directly to the researchers in a prepaid envelop, this risk was presumably minimized.

The explained variances in all the regression models were low, indicating that managers’ commitment and WOC only stand for a very limited part in the employees’ mental well-being and perception of how they well they perform their work. Regarding turnover intention, age seems to be more important than managers’ commitment as it added to the explained variance. The association between age and turnover intent was negative in slope which indicates younger persons to be more prone to leave their work than their older colleagues. This is in line with the results of Brewer *et al.* (2011) who showed that the younger generation of nurses was more interested in quitting from their current job than their older colleagues.

Possibly the perception of low QoC and the intention to leave could be due to low motivation in the employees. This may have been reflected in the responses but such attitudes may also increase the work load for the managers increasing the risk of managerial WOC. However, longitudinal studies are needed to understand the temporal relationship between these variables.

## Conclusions

In conclusion, the Spanish eldercare employees report better mental well-being, that they provide a better care quality and that they are less prone to leave their work place than Swedish eldercare workers. Moreover, the Spaniards are exposed to more committed and less work overcommitted managers than the Swedes. However, whereas this managerial exposure is associated with employees’ mental well-being, performance and wish to leave work in Spain, it is not in the Swedish employees. A probable explanation for this is the different preconditions, the managers have to deal with in the different countries.

This study contributes to the field by showing that eldercare employees fare better in a country and organization where managers are more committed to the organization but less overcommitted to work. Therefore, future research should focus on nursing managements’ conditions by taking into account the structure of the organizations. This is in line with Guerrero and Herrbach’s (2009) statement that the characteristics of the organization have attracted little attention in commitment research but is definitely needed.

## Figures and Tables

**Table I:** Background data on employees and comparisons between countries by *t*-test and *χ*^2^ tests

	Spain	Sweden	*t*(df)/*χ*^2^(df)	*p*
Age, *M* (SD)	43.77 (11.69)	44.12 (12.85)	*t*=0.30 (411)	0.78
Women, *n* (%)	227 (88)	154 (94)	*χ*^2^=2.53 (1)	0.11
Managers, *n* (%)	46 (15)	13 (7.2)	*χ*^2^=6.83 (1)	< 0.01

**Table II:** Differences between Spain and Sweden in means

	Spain *M* (SD)	Sweden *M* (SD)	*t*(df)	*p*
Managers’ commitment	3.84 (0.37)	3.37 (0.56)	10.07 (267)	<0.001
Managers’ overcommitment	2.74 (0.19)	3.49 (0.64)	12.46 (121)	<0.001
Employee well-being	1.78 (0.37)	1.96 (0.49)	3.91 (293)	<0.001
Employee quality of care	4.08 (0.63)	3.68 (0.71)	5.95 (327)	<0.001
Employee turnover intent	1.85 (1.12)	2.34 (1.36)	3.85 (302)	<0.001

**Table III:** Interaction and main effects of managers’ organizational commitment (CI) and work overcommitment (WOC) on employees’ mental well-being (GHQ12, please note that the scale is reverse), perception of quality of care (QoC) and turnover intent (TI)

	Employee GHQ12			Employee QoC			Employee TI		
	*β*	*p*	*R*^2^	*β*	*p*	*R*^2^	*β*	*p*	*R*^2^
*Managers’ commitment*									
CI×Country	0.08	0.05		−0.15	<0.05		0.38	<0.01	
CI	−0.16	<0.05		0.36	<0.001		−0.70	<0.001	
Country	0.11	<0.05	0.04	−0.12	0.05	0.07	0.24	<0.05	0.04
*Managers’ overcommitment*									
WOC×Country	−0.11	0.14		0.29	<0.05		−0.69	<0.001	
WOC	0.25	0.06		−0.66	<0.05		1.29	<0.01	
Country	0.07	0.16	0.04	0.06	0.40	0.07	0.16	0.21	0.03

**Notes:** Results by linear regression. *α* (*p*) for considering interaction set to 0.05

**Table IV:** Independent effects of managers’ organizational commitment and work overcommitment on employees’ mental well-being (GHQ12, please note that the scale is reverse), perception of quality of care (QoC) and turnover intent (TI) in Spain and Sweden, respectively

	Employee GHQ12			Employee QoC			Employee TI			
	*β*	*p*	*R*^2^	*β*	*p*	*R*^2^	*β*	*p*	*R*^2^	Adjusted
*Managers’ commitment*										
Spain	−0.08	<0.01	0.03	0.21	<0.001	0.06	−0.32	<0.001	0.04	
	−0.08	<0.01	0.03	0.20	<0.001	0.05	−0.30	<0.001	0.12	Sex, age
Sweden	−0.01	0.94	0.00	0.06	0.14	0.01	0.07	0.44	0.00	
	−0.01	0.80	0.01	0.06	0.15	0.01	0.03	0.75	0.13	Sex, age
*Managers’ work overcommitment*										
Spain				−0.37	<0.001	0.04	0.60	<0.01	0.03	
				−0.35	<0.001	0.03	0.60	<0.001	0.11	Sex, age
Sweden				−0.08	<0.05	0.03	−0.08	0.21	0.00	
				−0.08	<0.05	0.03	−0.12	<0.05	0.15	Sex, age

**Note:** Results from linear regression analyses

## References

[ref001] Aiken L., Sloane D., Bryneel L., Van den Heede K. and Sermeus W. (2013), “Nurses’ reports of working conditions and hospital quality of care in 12 countries in Europe”, International Journal of Nursing Studies, Vol. 50 No. 2, pp. 143-153. 23254247 10.1016/j.ijnurstu.2012.11.009

[ref002] Åkerstedt T., Nordin M., Alfredsson L., Westerholm P. and Kecklund G. (2012), “Predicting changes in sleep complaints from baseline values and changes in work demands, work control, and work preoccupation – the WOLF-project”, Sleep Medicine, Vol. 13, pp. 73-80. 22177346 10.1016/j.sleep.2011.04.015

[ref003] Astvik W. (2003), “Relationer som arbete. Förutsättningar för omsorgsfulla möten i hemtjänsten (Relations as work. Preconditions for caring encounters in home-help services)”, No. 2003:8. Thesis from Stockholm University, Elanders Gotab, Stockholm.

[ref004] Björk L., Bejerot E., Jacobshagen N. and Härenstam A. (2013), “I shouldn’t have to do this: illegitimate tasks as a stressor in relation to organizational control and resource deficits”, Work & Stress, Vol. 27 No. 3, pp. 262-277.

[ref005] Bolino M.C. and Turnley W.H. (2005), “The personal costs of citizenship behavior: the relationship between individual initiative and role overload, job stress, and work-family conflict”, Journal of Applied Psychology, Vol. 90 No. 4, pp. 740-748. 16060790 10.1037/0021-9010.90.4.740

[ref006] Brewer C.S., Kovner C.T., Greene W., Tukov-Shusner M. and Djukic M. (2011), “Predictors of actual turnover in a national sample of newly licensed registered nurses employed in hospitals”, Journal of Advanced Nursing, Vol. 68 No. 3, pp. 521-538. 22092452 10.1111/j.1365-2648.2011.05753.x

[ref007] Brown P., Fraser K., Wong C., Muise M. and Cummings G. (2013), “Factors influencing intentions to stay and retention of nurse managers: a systematic review”, Journal of Nursing Management, Vol. 21 No. 3, pp. 459-472. 23409964 10.1111/j.1365-2834.2012.01352.x

[ref008] Carney M. (2004), “Middle manager involvement in strategy development in not-for profit organizations: the director of nursing perspective-how organizational structure impacts on the role”, Journal of Nursing Management, Vol. 12 No. 1, pp. 13-21. 15101451 10.1111/j.1365-2834.2004.00388.x

[ref009] Clausen T., Bang Christensen K. and Nielsen K. (2015), “Does group-level commitment predict employee well-being? A prospective analysis”, Journal of Occupational and Environmental Medicine, Vol. 57 No. 11, pp. 1141-1145. 26539760 10.1097/JOM.0000000000000547

[ref010] Eddy P., Wertheim E.H., Hale M.W. and Wright B.J. (2018), “A systematic review and meta-analysis of the effort-reward imbalance model of workplace stress and hypothalamic-pituitary-adrenal axis measures of stress”, Psychosomatic Medicine, Vol. 80 No. 1, pp. 103-113. 28731983 10.1097/PSY.0000000000000505

[ref011] European Commission (2015), “Recruitment and retention of the health workforce in Europe”, Executive Summary, European Health Management Association, Brussels, April.

[ref012] Feldt T., Huhtala M., Kinnunen U., Hyvönen K., Mäkikangas A. and Sonnentag S. (2013), “Long-term patterns of effort-reward imbalance and over-commitment: investigating occupational well-being and recovery experiences as outcomes”, Work & Stress, Vol. 27 No. 1, pp. 64-87.

[ref013] Fisher D.M. (2014), “A multilevel cross-cultural examination of role overload and organizational commitment: investigating the interactive effects of context”, Journal of Applied Psychology, Vol. 99 No. 4, pp. 723-736. 24512027 10.1037/a0035861

[ref014] Glazer S. and Kruse B. (2008), “The role of organizational commitment in occupational stress models”, International Journal of Stress Management, Vol. 15 No. 4, pp. 329-344.

[ref015] Goldberg D.P. (1972), The Detection of Psychiatric Illness by Questionnaire: A Technique for the Identification and Assessment of Non-psychotic Illness, Oxford University Press, Oxford.

[ref016] Guerrero S. and Herrbach O. (2009), “Manager organizational commitment: a question of support or image?”, The International Journal of Human Resource Management, Vol. 20 No. 7, pp. 1536-1553.

[ref017] Guest D.E., Isaksson K. and De Witte H. (Eds) (2010), Employment Contracts, Psychological Contracts, and Employee Well-Being, Oxford University Press, Oxford.

[ref018] Gutermann D., Lehmann-Willenbrock N., Boer D., Born M. and Voelpel S.C. (2017), “How leaders affect followers work engagement and performance: integrating leader-member exchange and crossover theory”, British Journal of Management, Vol. 28 No. 2, pp. 299-314.

[ref019] Hofstede G. (1980), “Motivation, leadership, and organization: do American theories apply abroad?”, Organizational Dynamics, Vol. 9 No. 1, pp. 42-63.

[ref020] Hofstede G. (2001), Culture’s Consequences: Comparing Values, Behaviors, Institutions, and Organizations across Nations, 2nd ed., Sage, Thousand Oaks, CA.

[ref021] Hom P., Griffeth R. and Sellaro L. (1984), “The validity of Mobley’s (1977) model of employee turnover”, Organizational Behavior & Human Performance, Vol. 34 No. 2, pp. 141-174. 10268421 10.1016/0030-5073(84)90001-1

[ref022] Huang J., Wang Y., Wu G. and You X. (2016), “Crossover of burnout from leaders to followers: a longitudinal study”, European Journal of Work and Organizational Psychology, Vol. 25 No. 6, pp. 849-861.

[ref023] Meyer J. and Herscovitch L. (2001), “Commitment in the workplace: toward a general model”, Human Resource Management Review, Vol. 11 No. 3, pp. 299-326.

[ref024] Meyer J. and Maltin E. (2010), “Employee commitment and well-being: a critical review, theoretical framework and research agenda”, Journal of Vocational Behavior, Vol. 77 No. 2, pp. 323-337.

[ref025] Meyer J., Stanley D., Herscovitch L. and Topolnytsky L. (2002), “Affective, continuance, and normative commitment to the organization: a meta-analysis of antecedents, correlates, and consequences”, Journal of Vocational Behavior, Vol. 61 No. 1, pp. 20-52.

[ref035] Munduate L., García A.B., Pender E., Elgoibar P. and Medina F.J. (2015), “Employee representatives in Spain. Which are the perceptions and expectations by employers?”, in Euwema M. et al. (Eds), Promoting Social Dialogue in European Organizations. Industrial Relations and Conflict Management, Springer, London.

[ref026] Romeo M., Yepes M., Berger R., Guàrdia J. and Castro C. (2011), “Identification-commitment inventory (ICI model): confirmatory factor analysis and construct validity”, Qualitative Quantitative, Vol. 45, pp. 901-909.

[ref027] Rystedt L., Devereux J. and Sverke M. (2007), “Comparing and combining the demand-control-support model and the effort-reward imbalance model to predict long-term mental strain”, European Journal of Work and Organizational Psychology, Vol. 16 No. 3, pp. 629-653.

[ref028] Siegrist J. (2017), “The effort–reward imbalance model”, in Cooper C.L. and Quick J.C. (Eds), The Handbook of Stress and Health: A Guide to Research and Practice, John Wiley & Sons, Chichester, pp. 24-35.

[ref029] Siegrist J., Starke D., Chandola T., Godin I., Marmot M., Niedhammer I. and Peter R. (2004), “The measurement of effort-reward imbalance at work: European comparisons”, Social Science & Medicine, Vol. 58 No. 8, pp. 1483-1499. 14759692 10.1016/S0277-9536(03)00351-4

[ref030] Skakon J., Nielsen K., Borg V. and Guzman J. (2010), “Are leaders’ well-being, behaviours and style associated with the affective well-being of their employees? A systematic review of three decades of research”, Work & Stress, Vol. 24 No. 2, pp. 107-139.

[ref034] Skogstad A., Einarsen S., Torsheim T., Aasland M.S. and Hetland H. (2007), “The destructiveness of laissez-faire leadership behavior”, Journal of Occupational Health Psychology, Vol. 12 No. 1, pp. 80-92. 17257068 10.1037/1076-8998.12.1.80

[ref031] Wallin L., Pousette A. and Dellve L. (2014), “Span of control and the significance for public sector managers’ job demands: a multilevel study”, Economic and Industrial Democracy, Vol. 35 No. 3, pp. 455-481.

[ref032] Westerberg K. and Tafvelin S. (2014), “The importance of leadership style and psychosocial work environment to staff-assessed quality of care: implications for home help services”, Health and Social Care in the Community, Vol. 22 No. 5, pp. 461-468. 24313819 10.1111/hsc.12084

[ref033] Wong C. and Spence Laschinger H. (2015), “The influence of frontline manager job strain on burnout, commitment and turnover intention: a cross-sectional study”, International Journal of Nursing Studies, Vol. 52 No. 12, pp. 1824-1833. 26394531 10.1016/j.ijnurstu.2015.09.006

